# Hunger and its associated factors in the western Brazilian Amazon: a population-based study

**DOI:** 10.1186/s41043-022-00319-5

**Published:** 2022-08-17

**Authors:** Mayline Menezes da Mata, José Anael Neves, Maria Angélica Tavares de Medeiros

**Affiliations:** 1 Nutrition and Health, Health and Society Institute, Federal University of São Paulo (UNIFESP), Silva Jardim Street, 136, Vila Mathias, Santos, São Paulo, SP 11015-020 Brazil; 2 Federal University of São Paulo, 862, Botucatu Street, Vila Clementino, SP 04039-032 Brazil; 3 Health and Society Institute, Federal University of São Paulo (UNIFESP), Silva Jardim Street, 136, Vila Mathias, Santos, SP 11015-020 Brazil; 4 Department of Public Policies and Collective Health, Health and Society Institute, Federal University of São Paulo (UNIFESP), Silva Jardim Street, 136, Vila Mathias, Santos, SP 11015-020 Brazil

**Keywords:** Food and nutritional security, Nutrition programs and policies: nutritional epidemiology, Social inequality, Nutritional surveys

## Abstract

**Background:**

Hunger affects millions of people worldwide. In the current pandemic scenario of coronavirus Brazil has experienced an epidemic peak of hunger, amplifying existing prepandemic vulnerabilities, mainly in the North Region of the country. The aim of the present study was to investigate the prevalence of food insecurity and its associated factors in homes with children under 5 years of age in an urban area of a municipality of the western Brazilian Amazon.

**Methods:**

A household survey was conducted with a probabilistic sample of 557 children and their families. Food insecurity (FI) was determined using the Brazilian Food Insecurity Scale. Associations between variables were analyzed based on the prevalence ratio (PR) and respective 95% confidence intervals (CI) calculated through multiple Poisson regression analysis. Variables with a *P* value < 0.05 after adjustments were considered significantly associated with the outcome.

**Results:**

A prevalence of 76.5% (CI 1.36–2.67) food insecurity was found among the families in the study; 42.9% had moderate (CI 1.31–2.83) and severe (CI 1.10–1.83) food insecurity. Moderate and severe FI was associated with low family income (*P* = 0.00), participation in governmental income transfer programs (*P* = 0.01), and heads of household with less than 7 years of schooling (*P* = 0.02). Moreover, substantial frequencies of height deficit and being overweight were found among the children.

**Conclusions:**

The high prevalence of hunger and food insecurity and its associated factors reflects the context of geographic isolation and social exclusion in which these families live, suggesting that a substantial portion of the population under 5 years of age had experienced episodes of hunger in the 90 days prior to the survey. The prevalence of height deficit and being overweight among the children reveals a scenario of epidemiological/nutritional polarization, requiring the formulation of specific public policies for this population.

## Background

Hunger and food insecurity (FI) affects billions of people around the world. In recent years, efforts have been made to reduce the occurrence of FI. According to international agencies [1], Brazil is one of the 13 Latin American countries that were able to achieve a significant reduction in rates of hunger, malnutrition, and undereating in 2014.

However, hunger increased between 2015 and 2017, with more intense effects among populations with greater social vulnerability [2]. Due to its large size, Brazil still has deep social inequalities among the different geographic regions [3].

The North Region of Brazil has poor strategic development indicators, including a high rate of FI (57.0%), which is the highest in the country [4] and is associated with sociodemographic, economic, and environmental factors as well as access to public services [3]. Such negative indicators are accentuated in municipalities that are distant from capital cities which concentrate and monopolize supply systems and public services. As a result, more isolated municipalities suffer the most from the effects of social disparities [5].

Due to the current coronavirus disease 2019 (COVID-19) pandemic scenario, the state of Amazonas, in the North Region, has experienced the most severe health crisis in its history. In May 2020, its capital, Manaus, reached the epidemic peak, and in October of the same year, the attack rate by antibodies detectable (76%) exceeded the estimate for the state of São Paulo in the Southeast Region (29%) [6], the Brazilian state with the third highest population density in the country [7]. Consequently, in January 2021, during the second COVID-19wave [8], the Amazon health system collapsed. The efforts to mitigate the spread of the epidemic in Amazonas were insufficient, which led to an increase in both the mortality rate and pre-existing vulnerabilities, with negative impacts on social indicators all over the country [9].

At the present time, Brazil is experiencing an epidemic peak of hunger; approximately 19.1 million Brazilians are in severe food insecurity (IAG), and the North Region is one of the most affected, with an index corresponding to 18.1% of households in IAG [10].

The municipality of Coari is in the western portion of the Brazilian Amazon region and has the second highest gross domestic product in the state of Amazonas due to the export of petroleum and natural gas. Nevertheless, Coari has a low human development index (0.586) [11], and the child mortality rate is 18.2 per thousand live births [12], which demonstrates the precarious living conditions and health of the population. In the context of social inequities, it is essential to gain knowledge on the food and nutritional situation of homes in geographically isolated regions that are only accessible by water or air, which creates an obstacle for the execution of national surveys. The scarcity of scientific information on health, diet, and nutrition in the countryside of the state of Amazonas hinders planning and decision-making with regard to public policies to meet local needs.

The aim of the present study was to investigate the prevalence of food insecurity and its associated factors in homes with children under 5 years of age in a municipality of the western Amazon region of Brazil.

## Methods

### Design

A household survey was conducted in homes with children under 5 years of age in an urban area of the municipality of Coari in the state of Amazonas, Brazil, between March and July of 2018.

### Study setting

The municipality of Coari is located on the Urucu River of the Solimões River basin in the western portion of the Amazon region in Brazil. The city has an area of 58, 17.27 km^2^, is located 363 km from the state capital (Manaus), and is inaccessible by land. The estimated population in 2020 was 85,910 residents. In 2010, this population was 75,965: 49,651 in urban areas and 26,314 in rural areas, with a demographic density of 1.31 inhabitants per km^2^ [7].

### Sample size and sampling procedure

A probabilistic sample was performed. The sample size was calculated considering a population of 5903 children between 0 and 4 years of age in urban areas of the municipality [7], a 36.1% rate of FI in the North Region of the country in 2013 [13], a 95% confidence level, and a 4% absolute error. For the sample calculation, the following formula was used:

$$n = \frac{{N.z^{2} .p.q}}{{(N - 1).e^{2} + z^{2} .p.q}}$$

*N*—population size (5903); *z*^2^—standard distribution point (1.960); *p*—population proportion of the studied characteristic ⇒ *q* = 1 − *p* (0.36); *e*^2^—standard error (4.00%).

For the final sample, an adjustment was performed for a finite population, assuming a 10% dropout rate, which resulted in a minimum *n* of 506 + 10% = 557 children. A stratified random sampling technique was used. The total sample size was distributed proportionally to the 49 census tracts existing in the city, having as a sampling base the number of children between 0 and 4 years of age registered in the IBGE in each sector and the number of homes with at least one resident under 5 years old; a random drawing was performed, when necessary.

### Instrument and data collection

A questionnaire was created with closed-ended questions that were organized into the following components: identification/location of home; characterization of residents; characteristic of home; participation in income transfer programs; healthcare services; characteristics of the head of the family; characteristics of mother and child; and food insecurity. Interviews were held with the person in charge of the meals in the home. Twenty-two trained interviewers worked in pairs to conduct the interviews.

Food insecurity was assessed using the Brazilian Food Insecurity Scale (EBIA) [14], which enables measurement of families’ perceptions regarding access to food. The scale is composed of 14 items addressing the food situation experienced in the 90 days prior to the interview.

For the assessment of nutritional status, anthropometric measures were taken in duplicate using standard procedures and calibrated equipment. The following indices were considered: weight/age (W/A), height/age (H/A), weight/height (W/H), and body mass index/age (BMI/A). The indices were expressed as z-scores using the growth curves of the World Health Organization (WHO) as a reference [15].

The following variables of interest were used in the data analysis: demographic characteristics of the head of the household and children; 1. participation in governmental income transfer program; 2. characteristics of the home: number of rooms and existence of durable consumer goods; 3. occupation of head of family; 4. access to public services (water supply, garbage collection, electricity, and sewage system); 5. economic class according to the Brazilian Economic Classification Criteria [16] grouped into the following classes: A, B1, B2, C1, C2, D, and E; 6. child’s nutritional status; and 7. food insecurity.

### Data analysis

The data were double entered, and inconsistencies were verified with the aid of Epi Info®, version 7.0. Stata® version 13.0. (College Station, TX, USA) and WHO Anthro® (2005) version 3.2.2 (11) were used for the statistical analyses. Descriptive statistics were performed, with the calculation of absolute and relative frequencies for qualitative variables and measures of central tendency and dispersion for quantitative variables. Multiple Poisson regression analysis was performed to determine possible statistically significant associations between the response variable (moderate-to-severe food insecurity [MSFI]) and the predictors (covariables). PRs and respective 95% CIs were estimated in unadjusted (*p* < 0.20) and adjusted (*p* < 0.05) models. All variables with a *p* value < 0.20 in the univariate analysis were incorporated into the multiple regression model using the stepwise forwards procedure, and all variables with a *p value* < 0.05 after the adjustments remained in the final model as significantly associated with the outcome.

### Ethical aspects

This study received approval from the institutional review board of *Federal University of São Paulo* (certificate number: 83010018.8.0000.5505). Statements of informed consent and assessment (for mothers younger than 19 years of age) were read by the interviewers and presented for signatures. For those who reported having difficulties or did not know how to sign their names, an ink blotter was used to collect fingerprints.

## Results

From a total of 557 households with children selected in the sampling plan, the participation rate achieved was 100%. Among the 557 children under 5 years of age, 76.5% (95% CI 72.76–79.82%) had some degree of FI. Among these children, 33.58% (95% CI 29.75–37.61%) had mild FI, 17.4% (95% CI 14.47–20.80%) had moderate FI, and 25.5% (95% CI 22.03–29.29%) had severe FI, which meant that the children frequently experienced hunger.

Data on the sociodemographic characterization of the heads of household are displayed in Table 1. Among the 557 homes visited, 32.1% were headed by grandparents, 60.3% of heads of household were male, and the mean age was 40 years (standard deviation [SD]: 14.41). The majority had self-declared brown skin (71.8%), a spouse/partner (67.0%), up to 7 years of schooling (45.2%), no formal employment (75.58%) and earned the monthly minimum wage or less (67.5%), which was R$ 954 in 2018 (reference year of the study) [17].

**Table 1:** Sociodemographic characteristics of heads of households (*n* = 557) with children under 5 years of age in a municipality in the western Brazilian Amazon, 2019

Variables	*N*	%	95% CI
Sex			
Female	221	39.68	35.68–43.81
Male	336	60.32	56.18–64.31
Age group			
15–24 years	63	11.31	8.92–14.22
25–35 years	188	33.75	29.93–37.79
36–59 years	240	43.09	39.01–47.25
> 60 years	66	11.85	9.41–14.81
Skin color			
White	50	8.98	6.86–11.65
Non-White	507	91.02	88.34–93.13
Marital status			
With spouse	373	66.97	62.93–70.75
Without spouse	184	33.03	24.76–32.26
Schooling			
< 7 years	252	45.24	41.13–49.41
> 8 to < 14 years	213	38.24	34.28–42.36
> 15 years	92	16.52	13.65–19.84
Occupation—head of household			
Formal work	136	24.42	21.01–28.16
Informal work	421	75.58	71.83–78.98
Family income			
< monthly minimum wage	376	67.50	63.48–71.27
> monthly minimum wage	181	32.50	28.72–36.51

*CI* confidence interval. Monthly minimum wage: R$ 954 [12]

A total of 42.7% of the homes were made of wood and 98.4% had electricity, but only 21.9% had piped water from the public water supply, leading to a frequent lack of water (63.0%). Regarding the destination of domestic sewage, 45.0% of the residents dumped domestic sewage in the open air and in rivers/creeks adjacent to the home. A total of 50.8% of the homes were located on unpaved streets. The homes had an average of six residents. Economic classes D and E predominated (72.2%), and 78.3% of the interviewees reported participating in governmental income transfer programs.

Among the 557 children, 53.9% were boys, 79.5% had brown skin, and the mean age was 21 months (SD: 15.42). The prevalence of height for age deficits was 23.7% (95% CI 20.1–27.3%, SD: 1.39), and the prevalence of being overweight was 16.3% (95% CI 13.2–19.5%, SD: 1.22).

In the unadjusted Poisson regression analysis, MSFI was associated with the type of housing, number of residents, access to public garbage collection services, participation in governmental income transfer programs, and sociodemographic characteristics of the head of the family (Table 2).

**Table 2:** Unadjusted and adjusted models of associations between moderate to severe food insecurity and housing conditions, public services, sociodemographic characteristics of head of household, and height deficit per age in a municipality in the western Brazilian Amazon, 2019

Variables	Unadjusted model			Adjusted model		
	PR	CI (95%)	*p* value	PR	CI (95%)	*p* value
Home made of wood	1.27	0.97–1.67	0.07**	1.03	0.77–1.38	0.81
Not own home	1.08	0.77–1.52	0.62			
Number of residents in home > 4	1.32	0.90–1.94	0.15**	1.25	0.85–1.85	0.24
Water supply not public	1.17	0.85–1.62	0.32			
Lack of water in home	1.09	0.84–1.43	0.48			
Water not treated	1.17	0.90–1.51	0.22			
Trash not collected by public service	1.52	1.05–2.19	0.02*	1.13	0.76–1.69	0.52
Sewage deposited in open air	1.42	1.10–1.84	< 0.001*			
Street not paved	1.23	0.95–1.59	0.10**	1.11	0.84–1.47	0.43
Income						
< monthly minimum wage	2.13	1.53–2.95	< 0.001*	1.90	1.36–2.67	< 0.001*
Participation in income transfer programs	1.93	1.31–2.83	< 0.001*	1.63	1.10–2.42	0.01*
Sex						
Male	1					
Female	1.29	1.01–1.67	0.04*	1.22	0.94–1.58	0.12
Skin color						
White	1					
Non-white	1.47	0.87–2.48	0.14**	1.40	0.82–2.36	0.20
Marital status						
With spouse	1					
Without spouse	1.16	0.89–1.51	0.26			
Schooling						
< 7 years	1.41	1.10–1.83	< 0.001*	1.34	1.03–1.74	0.02*
> 8 years	1					
Occupation						
Formal	1					
Informal	1.56	0.85–2.85	0.14**	1.05	0.56–1.97	0.86
Height						
Adequate height for age	1					
Very low or low height for age	1.13	0.87–1.47	0.34			

The bold values highlight that they are statistically significant

*PR* prevalence ratio.* CI* confidence interval. Monthly minimum wage: R$ 954

*Statistically significant

**Criterion for inclusion in adjusted model (*p* < 0.20)

In the adjusted analysis, the following variables remained associated with MSFI: income, access to a governmental program, and schooling of the head of the family. Homes earning less than the monthly minimum wage were 1.90-fold more likely to experience episodes of MSFI than those that earned more than the monthly minimum wage (PR = 1.90; 95% CI 1.36–2.67). Families participating in governmental income transfer programs were 1.93-fold more likely to experience situations of MSFI than those who did not participate in such programs (PR = 1.93; 95% CI 1.31–2.83). Families in which the head of the household had a low level of schooling (≤ 7 years of study) were 1.41-fold more likely to experience MSFI than those in which the head of the household had a higher level of schooling (PR = 1.41; 95% CI 1.10–1.83).

**Fig. 1:**
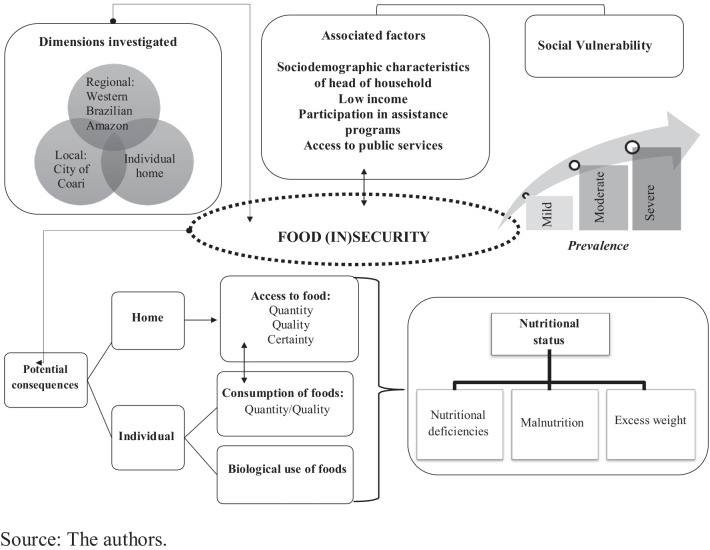
Conceptual model of determinants and potential consequences of food insecurity in a population of the western Brazilian Amazon, 2019

Figure 1 shows the conceptual model created to identify social determinants of IAG and potential consequences in this population.

## Discussion

A high rate of FI was found in homes with children under 5 years of age (36.7%), which was more than double the prevalence reported for homes with children under 5 years of age in Brazil as a whole [4]. Severe IAG affected 25.5% of the families interviewed, surpassing the prevalence reported for the North Region of the country (10.2%) and suggesting that a significant portion of the population under 5 years of age had suffered episodes of hunger in the 90 days prior to the interview. These results also surpass data from a recent Brazilian report, which points to the North Region as the most affected by hunger in the country [10]. In the same direction, the index of severe FI found in the present study exceeds the high prevalence identified in children from Ethiopia, a country that still lives with hunger and malnutrition in urban environments [18].

Among the characteristics analyzed, low income, participation in governmental income transfer programs, and schooling of the head of the household were associated with MSFI. These findings are compatible with data described by other authors [19].

An income below the monthly minimum wage (R$ 954) was the factor most associated with MSFI, implying difficulty in gaining access to food. The mean household income per capita in the municipality studied was R$ 347.20, with 62.4% of individuals earning less than R$ 140, which classifies these individuals as poor to extremely poor (IBGE 2010). This corresponds to half of the lowest mean per capita (R$ 605) recorded for Brazil in 2018, which was recorded in the state of Maranhão in the Northeast Region of the country [20]. Thus, the present results reaffirm findings in studies with a national scope, which state that a lower monthly household income is associated with a higher proportion of homes with FI, especially MSFI [19].

In the analysis of participation in governmental income transfer programs, which was also associated with MSFI, such programs are suggested to have achieved public priorities [21, 22]. Given the large portion of the interviewees in the present study who reported monetary transfers from the *Programa Bolsa Família* (Family Allowance Program) as the only source of income, FI would likely be greater without such programs.

Access to food is hindered by low income. Therefore, the association between MSFI and participation in programs such as the *Programa Bolsa Família* (Family Allowance Program) may be explained by the extreme poverty to which the heads of the households are submitted [23]. Although the trajectory of this program is related to a reduction in social inequalities in Brazil, structural disparities persist in the territories (regions, states, and municipalities). Income transfer alone is insufficient to interrupt poverty; in the sphere of public policies, it is also necessary to qualify services related to cross-compliance and expand intersectoral interventions targeting vulnerable populations [22, 24, 25].

Low levels of schooling, which was also associated with MSFI in the present study, make it difficult to enter the formal job market, implying an increase in informal (“under-the-table”) work and low pay, constituting a marker of inequity that favors the perpetuation of FI [19]. Regarding claims that the *Programa Bolsa Família* (Family Allowance Program) rectifies late enrollment, school attendance, and truancy rates of children from poorer families, there is evidence that these children leave school prematurely [26]. It should be stressed that an increase in the schooling of the population is a basic requirement to break free of the generational cycle of poverty [27].

As an aggravating factor of MSFI, a frequent lack of water was found in the homes due to low coverage by the public water supply [7] and the discarding of domestic sewage in the open air, rivers, and creeks adjacent to the homes. Although not part of the final statistical model, these aspects aggregate elements for the analysis of the vulnerability of the population. Even though Brazil is recognized for its considerable freshwater supply, part of the population does not have sufficient access to this water, especially in the North and Northeast Regions of the country [28]. Moreover, insufficient basic sanitation in the municipality of Coari, due to the natural dynamics of the waters (ebb and flow), could contribute to the maintenance or increase in child mortality rates, as limited access to water, sanitation, and adequate hygiene are among the determinants [29].

Height deficit was not associated with MSFI. Nonetheless, the high prevalence encountered (23.7%) is approximately double the average for the North Region (14.7%) (MS 2009) [30], demonstrating a situation of chronic malnutrition. This result is similar to the prevalence described in one of the least developed municipalities of Brazil, which is also located in the western Amazon Region (35.8%); the prevalence of a height deficit was associated with geographic isolation, social inequalities, and difficulties in gaining access to services in the North Region [31]. Regional data reveal that a height deficit in the population is the consequence of prolonged exposure to hunger and nutritional deficiencies [32], as children in northern Brazil are less likely to have access to a diversified, healthy diet than children in other regions of the country [33]. Despite the reduction in chronic malnutrition at the national level, regional inequities persist, which contribute to the high rates of childhood growth deficits in the North Region [34].

Along with the persistent problem of childhood malnutrition in the municipality, a substantial portion of the children studied was overweight, surpassing the global prevalence estimated for children and adolescents reported by a study that found a growing trend of excess weight in developing countries (from 8.1 to 12.9% among males and from 8.4 to 13.4% among females). These rates are higher in developed countries (23.8% among males and 22.6% among females) [35]. Children from families with FI in developed countries also have a greater likelihood of poorer health since birth and a worse perception of their health on the part of their parents [36].

No association between MSFI and being overweight was found in the present study, which is similar to findings in an investigation involving data from the Brazilian Demographics and Health Survey [30, 37]. However, Tarasuk et al. [38] suggest that MSFI may be a conditioning factor for an increase in morbidity rates and hospitalizations. Likewise, Gomes et al. [39] found that nutritional risk among individuals with chronic morbidities was greater among those with MSFI. Moreover, excess weight in childhood is a risk factor for obesity and chronic noncommunicable diseases in adulthood [40].

The context of inequities in the families that participated in the present study was characterized by the low economic status and schooling of the heads of the households, massive participation in governmental income transfer programs, and inadequate housing and sanitation, painting a picture of intense social disparities.

MSFI and associated factors reveal the precariousness of economic development and public policies in areas distant from large metropolises. The geographic isolation of the region is a recurrent justification for the historical lack of public policies directed at this population. As a result, social exclusion deepens, which is evidenced in lower income strata and in people with lower levels of schooling being deprived of access to the job market and public services [41]. The intensification of these social disparities, mainly in territories historically marked by social inequalities, is among the impacts of the pandemic by the new COVID-19. Thus, it is suggested that moderate and severe FI is currently even higher than they were historically [42]. The state should play a central role in the fulfillment of basic rights that favor the social inclusion of the population and the consequent overcoming of socioeconomic inequalities and FI. Likewise, social control agencies should perform a role in monitoring this process. However, Brazil is currently passing through a period marked by austerity policies, which weaken existing social protection policies [43]. It is, therefore, necessary to place FI and the human right to adequate, healthy food on the agenda of different governmental courts of Brazil for the enforcement of public policies for combating hunger and poverty [44].

A limitation of the present study was the impossibility of including rural areas in the survey due to budgetary and logistic deficiencies. Otherwise, the research's innovativeness resides in the fact that it is an unprecedented population-based inquiry addressing a remote municipality in the western portion of the Brazilian Amazon region that is not covered in national surveys. Therefore, the present investigation fills a gap in knowledge on this issue regarding municipalities in the countryside of northern Brazil.

## Conclusions

The high prevalence of hunger and FI associated with low income, low schooling of the heads of households, and participation in governmental income transfer programs translates to the structural vulnerability of these families. Moreover, the substantial height deficit and simultaneous prevalence of overweight children point to epidemiological-nutritional polarization in this municipality of the western Brazilian Amazon. Geographic isolation and limited access to public services are suggested to exert a negative impact on social inclusion in this population, contributing to the perpetuation of inequalities.

## Data Availability

The datasets used and/or analyzed during the current study are available from the corresponding author on reasonable request.

## Abbreviations

FI: Food insecurity
W/A: Weight/age
H/A: Height/age
W/H: Weight/height
BMI/A: Body mass index/age
MSFI: Moderate to severe food insecurity
PR: Prevalence ratios
CI: Confidence intervals
SD: Standard deviation
